# Cause-Specific Mortality Among Survivors From T1N0M0 Renal Cell Carcinoma: A Registry-Based Cohort Study

**DOI:** 10.3389/fonc.2021.604724

**Published:** 2021-03-10

**Authors:** Zhixian Wang, Jing Wang, Yunpeng Zhu, Chang Liu, Xing Li, Xiaoyong Zeng

**Affiliations:** ^1^ Department of Urology, Tongji Hospital, Tongji Medical College, Huazhong University of Science and Technology, Wuhan, China; ^2^ Department of General Medical, Tongji Hospital, Tongji Medical College, Huazhong University of Science and Technology, Wuhan, China; ^3^ Institute of Urology of Hubei Province, Wuhan, China

**Keywords:** renal cell carcinoma, competing mortality, standardized mortality ratio, long-term survival, prognosis

## Abstract

**Objective:**

More T1N0M0 renal cell carcinoma (RCC) is detected and the prognosis has improved, but, the current focus on non-RCC-related mortality is superficial. We investigated cause-specific mortality and its temporal patterns after an RCC diagnosis.

**Methods:**

In the Surveillance, Epidemiology, and End Results-18 database, patients with T1N0M0 RCC treated with partial nephrectomy (PN) or radical nephrectomy (RN) during 2000–15 were identified. Standardized mortality ratios (SMRs) for cause of death were calculated. Risk predictors for each cause-specific mortality were investigated using the Fine and Gray sub-distribution model.

**Results:**

In all, 68,612 eligible patients were pooled. A total of 14,047 (20.5%) patients had died (cardiovascular disease [CVD], 28.3%; other non-cancer-related diseases, 20.3%; RCC, 18.7%; other cancer types, 16.3%; non-disease events, 16.1%) during follow-up. Heart disease, diabetes mellitus, and cerebrovascular disease were the primary causes of non-RCC-related mortality within 1 year after the diagnosis. The greatest proportion of death (39.0%) occurred within 1–5 years after the diagnosis, mostly due to RCC itself, followed by heart disease. However, >5 years after the diagnosis, heart disease became the leading cause of death. Compared with the general US population, a 21% (SMR, 1.21; 95%CI 1.19–1.23) increased risk of all-mortality was observed; RCC patients had a higher risk of heart disease-related death within 5–10 years (SMR, 1.10; 95%CI 1.04–1.17) and >10 years (1.12; 1.02–1.22) after the diagnosis. Older age and RN increased the death risk of CVD and RCC-specific mortality. Although a larger tumor diameter increased the risk of RCC-specific death, this was not a significant predictor for CVD. Moreover, for T1N0M0 RCC tumors of diameter >4 cm, there was no significant difference in CVD incidence for RN *vs.* PN.

**Conclusions:**

RCC-specific mortality is a common challenge for the prognosis. Importantly, a large proportion and higher SMRs of other non-RCC-related diseases (especially CVD) should not be disregarded for the better holistic management of survivors of local RCC. Targeted prevention strategies for non-RCC-related death could lead to significant reductions in mortality for RCC survivors.

## Introduction

Due to the frequency of abdominal imaging, many local small renal cell carcinomas (RCCs) are detected incidentally. Upon combination with partial nephrectomy (PN) or radical nephrectomy (RN) as curative surgery, healthcare support, and meticulous postoperative surveillance, the prevalence of survival from RCC has improved substantially in recent decades (1, 2). Five-year RCC-specific survival can reach >90% after surgery for non-metastatic small RCC (3–7). There has been a decrease in mortality prevalence due to RCC itself (8) and patients can expect long-term survival (5–7, 9). Nevertheless, a considerable number of RCC patients may die of treatment-related or other causes, which threatens overall survival (OS) despite the improvement in RCC-specific survival (10–12). Therefore, investigating other mortality risks which challenge long-term OS after RCC patients have undergone RN or PN is crucial, as is provision of better counseling regarding future health risks in RCC survivors.

Here, we present the most recent registry-based [Surveillance, Epidemiology, and End Results-18 (SEER-18) database] long-term analyses of the specific causes of mortality and its temporal patterns among patients with T1N0M0 RCC. We analyzed its associated risks compared with the general population of the USA by adjusting for age and duration of follow-up. Besides, we investigated possible associations between different demographic- and RCC-related characteristics with the risk of each cause-specific mortality in patients with T1N0M0 RCC.

## Patients and Methods

### Design and Data Sources

In our registry-based retrospective, observational cohort study, we accessed the SEER-18 database (study approval username: 10062-Nov2019). SEER*Stat 8.3.6.1 was used for selection and analyses of case lists. The SEER program from the National Cancer Institute (Bethesda, MD USA) is a widely used and comprehensive source of data on the incidence, staging, and treatment of cancer, as well as cancer survival. To avoid a surveillance bias, quality assurance was ensured by systematic, standardized, and periodic procedures for data collection. The SEER-18 database covers ~28% of the USA population, with clinical and demographic characteristics that are comparable with those of the general population (https://seer.cancer.gov/). SEER data are anonymized, so approval from the ethics committee of our institution was waived.

### Population Identification

**Supplementary Figure 1** shows the flowchart for data selection from the SEER database. Patients who were >14 years of age with a diagnosis of localized first primary RCC between 2000 and 2015, and tumor diameter <7 cm (equivalent to clinical T1N0M0 RCC) treated by PN or RN were included for evaluation. The stage of localized RCC was identified according to historic SEER stage A (applicable between 1973 and 2015), which categorizes cancers into “localized”, “regional”, or “distant” disease for specific malignancies. To control for a selection bias, we included all eligible cases. We did not include patients who did not undergo surgery or who underwent non-PN or non-RN (e.g., radiofrequency ablation, cryoablation) because most of this population was older and had comorbidities, which increases the risk of non-RCC death.

### Study Variables

We selected a range of demographic and clinical variables: year of diagnosis; marital status; age at the diagnosis; sex, race/ethnicity [white, black, and others (American Indian/Alaska Native, Asian Native, and Asian/Pacific Islander)]; Tumor-related characteristics were recorded: tumor diameter (cm) and histology cell type for RCC (clear cell, papillary, chromophobe, and other or undefined cell types); tumor grade [“well differentiated” (grade I), “moderately differentiated” (grade II), “poorly differentiated” (grade III), and “undifferentiated” (grade IV)]; tumor laterality; and treatment (PN or RN). We inspected cause-specific mortality with respect to the abovementioned variables, and each according to the latency period (<1, 1–5, 5–10, and >10 years after the RCC diagnosis).

### Outcomes

The cause of death was obtained from the SEER Cause of Death Code, which was identified according to the 10th edition of the *International Statistical Classification of Diseases and Related Health Problems* (**Supplementary Table 1**). In our study, the primary outcomes for analysis were stratified as cardiovascular disease [(CVD), including heart disease, hypertension without heart disease, cerebrovascular disease, atherosclerosis, or aortic aneurysm], other non-cancer-related diseases (diseases except for cancer and CVD), RCC, other cancer-related diseases (all cancers except RCC), and non-disease events (accidents and adverse effects, suicide and self-inflicted injury, homicide and legal intervention, and others).

### Study Objectives

The objectives of our study were to: i) determine the proportion of deaths based on different causes, temporal patterns, and treatment after a first diagnosis of primary T1N0M0 RCC; ii) assess the risk of a specific cause of death using standardized mortality ratios (SMRs); iii) investigate the risk factors for each cause-specific mortality after PN or RN among patients with T1N0M0 RCC.

### Statistical Analyses

Distribution of all demographic and clinical characteristics is described using the mean [standard deviation (SD)] or median [interquartile range (IQR)] for continuous variables and frequency (%) for categorical variables. We reported the percentages of deaths among patients with RCC within each time period. We reported the percentages of each cause of death among deaths from RCC within each time period. Crude cumulative incidence functions were used to calculate and plot cumulative cause-specific mortality, overall mortality, and mortality stratified by treatment, age, and tumor diameter to describe the probability of experiencing a specific event in the presence of competing risks among patients with T1N0M0 RCC treated by PN/RN. The SMR is calculated as the observed-to-expected ratio. “Observed” represented the number of RCC patients who died from a specific cause of mortality during the follow-up period. “Expected” represented the number of people expected to die by the same specific cause of death in a demographically similar population within the same timeframe and with consideration of the year of the RCC diagnosis (13). Here, the SMR represented the change in the risk for a specific cause of death after a RCC diagnosis compared with the risk for the general population of the USA. Mortality data for the general USA population were collected and maintained by the National Center for Health Statistics, and were retrieved and analyzed using SEER*Stat. The data include all causes of death, not just cancer-related deaths, and the National Center for Health Statistics granted the SEER database limited permission to provide mortality data to the public. By using SEER*Stat, we calculated all SMRs with 95% confidence intervals (CIs), and *P*-values based on 95% CIs were estimated according to the method described by Altman and colleagues (14). Age-specific SMRs were calculated according to stratified age groups (<49, 50–59, 60–69, and 70+ years of age) and stratified by PN and RN. Duration of follow-up (<5, 5–10, and ≥10 years) also impacts event occurrence. Treatment- and time interval-adjusted SMRs were calculated to assess the effects on the analyzed outcomes. Finally, adjusted Fine and Gray sub-distribution hazards models were used to estimate the prognostic predictors by hazard ratio (HR) on different cause-specific mortalities (RCC, CVD, other non-cancer-related diseases, other types of cancer, and other non-disease events). Cox proportion risk regression was employed to predict all-cause mortality. In the Fine and Gray model, each specific cause of mortality was included as an endpoint, and other causes of death were regarded as competing events in the model. All analyses were conducted using R v.3.6.3 (R Foundation for Statistical Computing, Vienna, Austria; www.r-project.org/). *P* < 0.05 (two-sided) was considered significant.

## Results

### Baseline Characteristics

**Table 1** lists the characteristics of the study cohort diagnosed with RCC between 2000 and 2015. In total, 68,612 RCC patients were eligible for inclusion in our study. The median age of the cohort at the diagnosis was 60 [IQR: 51–68] years (data not shown); 55.6% were aged 50–69 years, and 21.9% were aged >70 years. Most RCC patients were white (81.4%), male (60.4%), married (62.7%), had clear cell RCC (56.9%), and had undergone RN (59.2%).

**Table 1:** Baseline characteristics of all patients with renal cell carcinoma and patients who died according to the causes of mortality.

	Total No. of Patients (% of each group)			Causes of mortality										
		All Deaths		Renal cell carcinoma		Cardiovascular disease		Other non-cancer diseases		Other cancers		Non-diseases cause		
		No. ofPatients (%^a^)	Mean Age at Death, y	No. ofPatients (%^a^)	Mean Age at Death, y	No. ofPatients (%^a^)	Mean Age at Death, y	No. ofPatients (%^a^)	Mean Age at Death, y	No. ofPatients (%^a^)	Mean Age at Death, y	No. ofPatients (%^a^)	Mean Age^a^ at Death, y	
**Overall cohort**	68612	14047 (20.5)	72.5	2633 (18.7%)	69.7	3984 (28.4%)	74.4	2862 (20.4%)	72.9	2299 (16.4%)	72.7	2269 (16.2%)		71.9
**Year group of diagnosis**														
2000–2004	15633 (22.8%)	6594 (42.1)	74.2	1161 (17.6%)	70.3	1921 (29.1%)	76.5	1381 (20.9%)	74.8	1123 (17.0%)	73.9	1008 (15.3%)		73.9
2005–2009	21972 (32.0%)	5136 (23.1)	72.1	983 (19.1%)	70.1	1389 (27.0%)	73.7	1053 (20.5%)	72.3	837 (16.3%)	71.9	874 (17.0%)		71.8
2010–2015	31007 (45.2%)	2317 (7.1)	68.6	489 (21.1%)	67.7	674 (29.1%)	69.6	428 (18.5%)	68.2	339 (14.6%)	70.3	387 (16.7%)		67.1
**Age group of diagnosis**														
≤49 years	15446 (22.5%)	1160 (8.1)	49	271 (23.4%)	48.6	239 (20.6%)	50.2	232 (20.0%)	49.1	145 (12.5%)	50.5	273 (23.5%)		47.6
50–59 years	18649 (27.2%)	2516 (13.1)	61	579 (23.0%)	60.6	628 (25.0%)	61.1	516 (20.5%)	60.8	424 (16.9%)	61.9	369 (14.7%)		60.5
60–69 years	19466 (28.4%)	4026 (21.1)	70.5	816 (20.3%)	69.7	1072 (26.6%)	70.6	799 (19.8%)	70.7	740 (18.4%)	70.9	599 (14.9%)		70.8
70+ years	15051 (21.9%)	6345 (42.1)	82.7	967 (15.2%)	81.2	2045 (32.2%)	83.3	1315 (20.7%)	83.2	990 (15.6%)	81.8	1028 (16.2%)		83.2
**Race^b^**														
White	55840 (81.4%)	11464 (21.1)	73.4	2216 (19.3%)	70.3	3174 (27.7%)	75.6	2272 (19.8%)	74.1	1935 (16.9%)	73	1867 (16.3%)		72.6
Black	8180 (11.9%)	1891 (23.1)	67.9	268 (14.2%)	65.8	638 (33.7%)	68.4	469 (24.8%)	67.6	243 (12.9%)	69.7	273 (14.4%)		67.7
Other	4056 (5.9%)	678 (17.1)	71.6	149 (22.0%)	68.5	166 (24.5%)	74.2	117 (17.3%)	71.3	119 (17.6%)	73	127 (18.7%)		71
**Sex**														
Female	27138 (39.6%)	5254 (19.1)	74.7	929 (17.7%)	71.5	1511 (28.8%)	77	1184 (22.5%)	75.1	783 (14.9%)	73.6	847 (16.1%)		74.4
Male	41474 (60.4%)	8793 (21.1)	71.3	1704 (19.4%)	68.8	2473 (28.1%)	72.8	1678 (19.1%)	71.3	1516 (17.2%)	72.2	1422 (16.2%)		70.4
**Marital status^b^**														
Never married	10162 (14.8%)	1791 (18.1)	65	306 (17.1%)	63.9	490 (27.4%)	66.3	409 (22.8%)	64.2	253 (14.1%)	67.9	333 (18.6%)		63
Married	43023 (62.7%)	7949 (18.1)	72.8	1616 (20.3%)	69.5	2155 (27.1%)	74.7	1505 (18.9%)	73.6	1415 (17.8%)	72.7	1258 (15.8%)		72.8
Widowed/Divorced/Separated	12229 (17.8%)	3765 (31.1)	75.8	619 (16.4%)	73.5	1187 (31.5%)	77.3	840 (22.3%)	75.8	541 (14.4%)	75.2	578 (15.4%)		75.8
**Histology of RCC**														
ccRCC	39015 (56.9%)	6907 (18.1)	72.2	1390 (20.1%)	69.9	1978 (28.6%)	74.2	1335 (19.3%)	72.5	1105 (16.0%)	72.5	1099 (15.9%)		70.8
paRCC	8390 (12.2%)	1606 (19.1)	71.2	217 (13.5%)	70.3	478 (29.8%)	71	383 (23.8%)	71	272 (16.9%)	71.6	256 (15.9%)		72.1
chRCC	3719 (5.4%)	470 (13.1)	75.9	52 (11.1%)	72.3	150 (31.9%)	77.6	102 (21.7%)	76.7	76 (16.2%)	76.8	90 (19.1%)		73.5
undefinedRCC	13758 (20.1%)	3966 (29.1)	73.1	730 (18.4%)	69.8	1089 (27.5%)	75.1	837 (21.1%)	73.7	636 (16.0%)	72.8	674 (17.0%)		73
Other	3730 (5.4%)	1098 (29.1)	73.1	244 (22.2%)	67.7	289 (26.3%)	76.7	205 (18.7%)	73.8	210 (19.1%)	73.1	150 (13.7%)		73.8
**Grade of RCC**														
I+II	43437 (63.3%)	8136 (19.1)	72.7	1217 (15.0%)	71.1	2423 (29.8%)	74.2	1739 (21.4%)	72.7	1371 (16.9%)	72.4	1386 (17.0%)		71.6
III+IV	14147 (20.6%)	3155 (22.1)	71.4	945 (30.0%)	68	712 (22.6%)	73.8	590 (18.7%)	72.4	458 (14.5%)	73.2	450 (14.3%)		71.2
Unknown	11028 (16.1%)	2756 (25.1)	73.5	471 (17.1%)	69.8	849 (30.8%)	75.5	533 (19.3%)	73.8	470 (17.1%)	73	433 (15.7%)		73.6
**Laterality b**														
Left	33129 (48.3%)	6806 (21.1)	72.5	1287 (18.9%)	69.8	1883 (27.7%)	74.2	1377 (20.2%)	73	1112 (16.3%)	72.9	1147 (16.9%)		72
Right	35466 (51.7%)	7236 (20.1)	72.5	1344 (18.6%)	69.7	2101 (29.0%)	74.6	1484 (20.5%)	72.8	1186 (16.4%)	72.4	1121 (15.5%)		71.9
**Surgery**														
Partial nephrectomy	27985 (40.8%)	3135 (11.1)	71.2	427 (13.6%)	69.6	950 (30.3%)	72.9	604 (19.3%)	71.3	590 (18.8%)	71.8	564 (18.0%)		68.8
Radical nephrectomy	40627 (59.2%)	10912 (27.1)	72.9	2206 (20.2%)	69.8	3034 (27.8%)	74.9	2258 (20.7%)	73.3	1709 (15.7%)	73	1705 (15.6%)		73
**Tumor size group**														
~ 2 cm	11944 (17.4%)	1740 (15.1)	70.6	155 (8.9%)	69	549 (31.6%)	72.1	414 (23.8%)	69.7	292 (16.8%)	71.5	330 (19.0%)		69.2
2–3 cm	16863 (24.6%)	2917 (17.1)	72.8	325 (11.1%)	72.6	911 (31.2%)	73.7	655 (22.5%)	72.7	513 (17.6%)	73.5	513 (17.6%)		71
3–4 cm	15006 (21.9%)	3153 (21.1)	73.2	460 (14.6%)	71.1	964 (30.6%)	74.5	683 (21.7%)	73.2	536 (17.0%)	72.6	510 (16.2%)		73.1
4–5 cm	11454 (16.7%)	2697 (24.1)	72.9	574 (21.3%)	69.5	720 (26.7%)	75.8	535 (19.8%)	73.8	441 (16.4%)	72.3	427 (15.8%)		72.4
5–7 cm	13345 (19.4%)	3540 (27.1)	72.3	1119 (31.6%)	68.6	840 (23.7%)	75.2	575 (16.2%)	74.2	517 (14.6%)	72.9	489 (13.8%)		73.2
**Second primary malignancy onset**														
Yes	17396 (17.7%)	3640 (21.1)	73.2	471 (12.9%)	71.5	562 (15.4%)	74.9	393 (10.8%)	74.8	1887 (51.8%)	72.5	327 (9.0%)		74.8
No	81113 (82.3%)	10407 (13.1)	72.3	2162 (20.8%)	69.4	3422 (32.9%)	74.3	2469 (23.7%)	72.6	412 (4.0%)	73.2	1942 (18.7%)		71.4

ccRCC, clear cell renal cell carcinoma; paRCC, papillary renal cell carcinoma; chRCC, chromophobe renal cell carcinoma.

a, % of total death.; b, not present the information of unknown race (n = 536), unknown the Marital status (n = 3198), and unknown laterality (n = 17).

### Outcomes and Causes of Mortality

Up to the final follow-up, 14,047 patients (20.5%) had died, and the mean age at death was 72.5 years (**Table 1**). In total, among all causes of death, 2,633 (18.7%) deaths were RCC-specific with mean 69.7 years old at death, 28.4% were due to CVD with mean 74.4 years old at death, 20.4% were due to other non-cancer-related diseases with mean 72.9 years old at death, 16.4% were due to other types of cancer, and 16.2% were from non-disease events (**Table 1** **)**.

**Figure 1:**
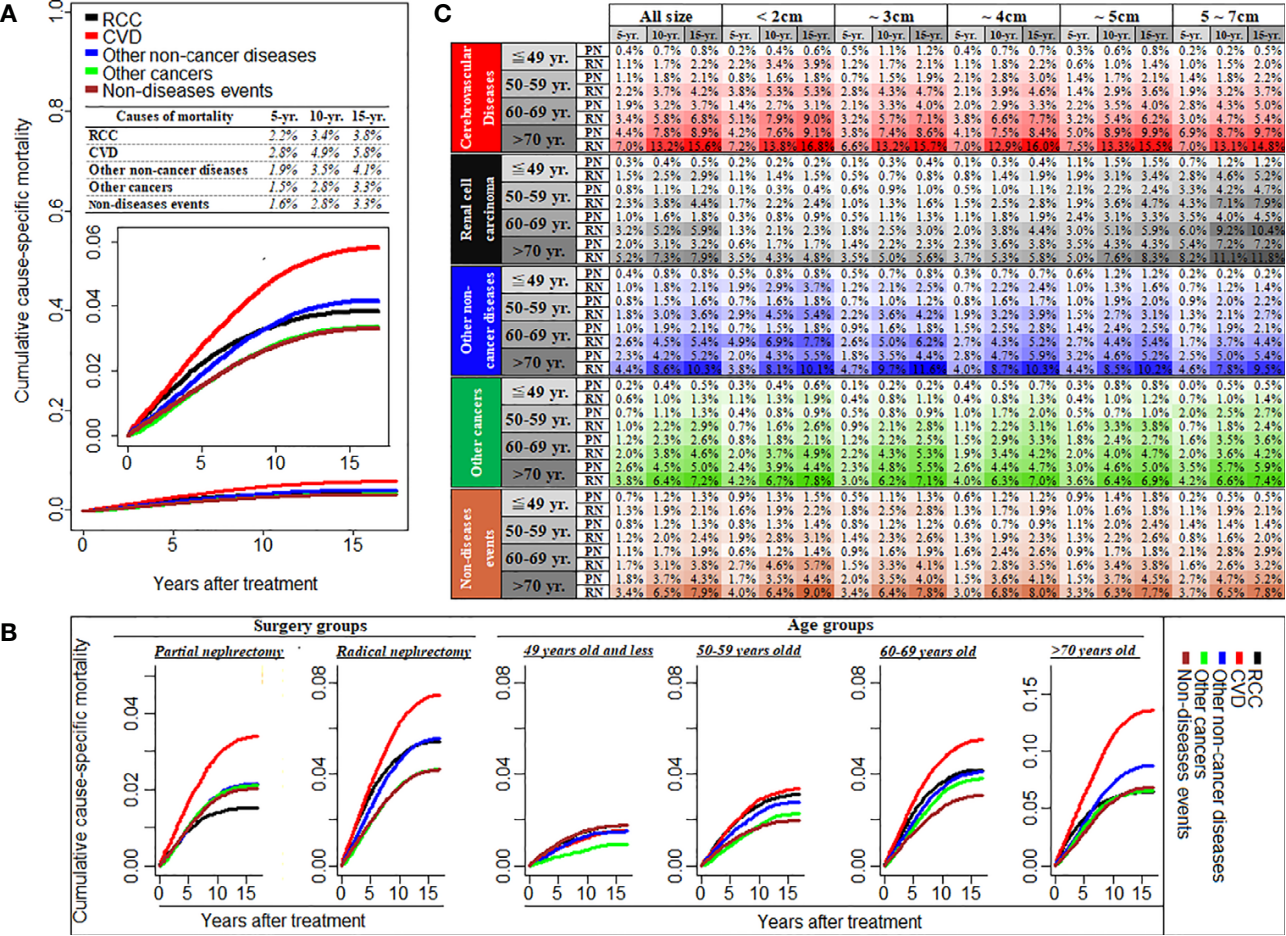
Cumulative cause-specific mortality among T1N0M0 renal cell carcinoma patients **(A)**, and stratified by the treatment of partial or radical nephrectomy and different age groups **(B)**, and also stratified by tumor size groups with the subgroup of different age and treatment **(C)**. RCC, renal cell carcinoma; CVD, Cardiovascular disease; PN, partial nephrectomy; RN, radical nephrectomy; CVD including heart disease, hypertension, cerebrovascular disease, and atherosclerosis/aortic aneurysm and Dissection.

**Figure 1** shows cumulative cause-specific mortality among T1N0M0 RCC patients and stratified by treatment (PN or RN), age group, and tumor diameter. Cumulative mortality was highest for CVD, with a prevalence of 2.8% and 4.9% at 5 and 10 years, respectively, which was higher than that for RCC-specific mortality (**Figure 1A**). Compared with RN patients, the cumulative mortality incidence by CVD or RCC in patients treated by PN was lower. Cumulative mortality by CVD increased with increasing age, and became the main cause of death in patients aged >50 years, and particularly so in those aged >60 years, at the diagnosis of RCC (**Figure 1B** detail mortality rate data showed in **Supplementary Table 2**). Interestingly, after adjustment of tumor diameter according to age, the cumulative incidence of CVD mortality was highest among RCC patients with tumor diameter <2 cm compared with those with tumor diameter ≥2 cm, and this increased with age. In patients aged ~49 years to >70 years with tumor diameter <2 cm, the CVD prevalence ranged from 3.95% to 16.8% in patients treated by RN, compared with 0.6%–9.1% in patients treated by PN (**Figure 1C**). Furthermore, we found that 76.2% CVDs were due to heart diseases, and respiratory/digestive system cancer death (54.7%) were the common cause of mortality due to other non-RCC cancer, besides, nephritis, nephrotic syndrome and nephrosis and diabetes mellitus were the common other non-cancer diseases (**Supplementary Table 3**).

**Figure 2:**
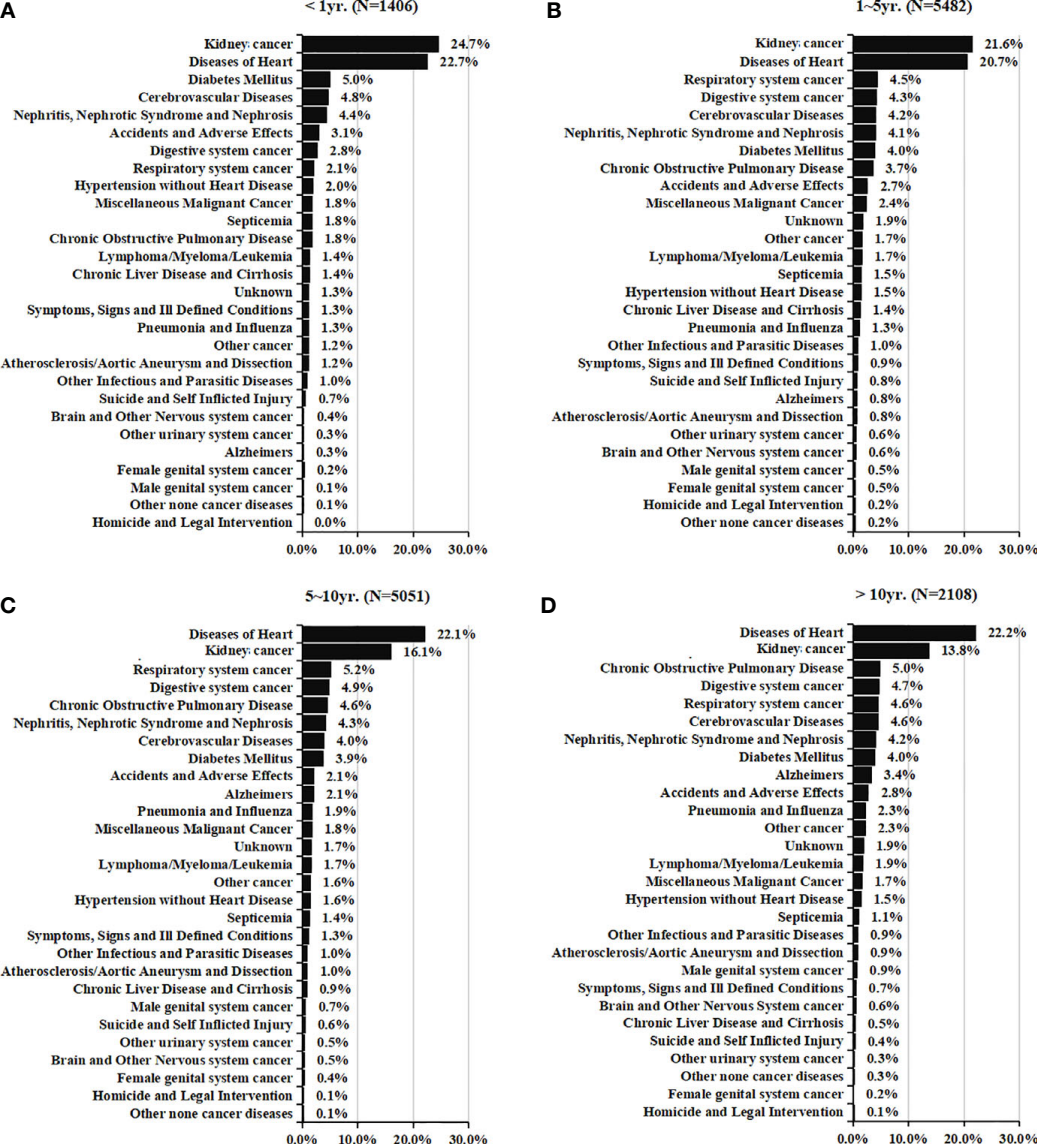
Causes of death in each latency period of < 1 year **(A)**, 1-5 years **(B)**, 5-10 years **(C)**, and > 10 years **(D)** following T1N0M0 renal cell cancer diagnosis.

**Figure 2** showed the causes of death in different latency period following RCC treatment. In total, 1406 deaths (10.0%, **Table 2**) occurred within <1 year after the RCC diagnosis, and RCC-specific death remained the leading cause of mortality during this period. Heart diseases, diabetes mellitus, cerebrovascular disease, nephritis, and accidents were the common causes of non-RCC mortality within 1 year after the diagnosis. The highest number of deaths (5842; 39.0%, **Table 2**) occurred within 1–5 years after the RCC diagnosis, and RCC was the most common cause of mortality, followed by heart disease. Also, 5051 (36.0%, **Table 2**) deaths occurred within 5–10 years, and 2,108 (15.0%, **Table 2**) deaths occurred at >10 years, after the RCC diagnosis. In patients surviving >5 years, heart disease was the leading cause of death, death from RCC itself was the second most prevalent cause of mortality, and chronic obstructive pulmonary disease was the second most common non-cancer-related cause of mortality. In addition, mortality due to cancer of respiratory or digestive systems was the primary non-RCC cancer-related cause of death during the entire duration of follow-up.

**Table 2:** Baseline characteristics of all patients with renal cell carcinoma and patients who died according to the time of death after diagnosis.

	Timing of Deaths After Diagnosis								
	<1 year		1 to 5 years		5 to 10 years		≥10 years		
	No. of Patients (%^a^)	Mean Age at Death, y	No. of Patients (%^a^)	Mean Age at Death, y	No. of Patients (%^a^)	Mean Age at Death, y	No. of Patients (%^a^)	Mean Age at Death, y	
**Overall cohort**	1406 (10.0%)	67.9	5482 (39.0%)	69.8	5051 (36.0%)	74.5	2108 (15.0%)	77.9	
**Year group of diagnosis**									
2000–2004	452 (6.9%)	69.2	1849 (28.0%)	70.6	2380 (36.1%)	74.9	1913 (29.0%)	77.9	
2005–2009	445 (8.7%)	67.3	2042 (39.8%)	69.9	2454 (47.8%)	74.4	195 (3.8%)	68.6	
2010–2015	509 (22.0%)	67.2	1591 (68.7%)	68.7	217 (9.4%)	71.4	–	–	
**Age group of diagnosis**									
≤49 years	111 (9.6%)	43.5	481 (41.5%)	46.2	382 (32.9%)	51.2	186 (16.0%)	55.2	
50–59 years	247 (9.8%)	55.6	994 (39.5%)	58.2	876 (34.8%)	62.6	399 (15.8%)	68.7	
60–69 years	401 (10.0%)	65.5	1577 (39.2%)	67.8	1431 (35.5%)	72.1	617 (15.3%)	78.5	
70+ years	647 (10.2%)	78.2	2430 (38.3%)	80.5	2362 (37.2%)	84.2	906 (14.2%)	89.1	
**Race^b^**									
White	1080 (9.4%)	68.6	4386 (38.3%)	70.7	4235 (36.9%)	75.2	1763 (15.4%)	78.5	
Black	249 (13.2%)	65	827 (43.7%)	65.2	572 (30.2%)	70.1	243 (12.9%)	72.7	
Other	75 (11.1%)	66.7	261 (38.5%)	68.9	241 (35.5%)	73.6	101 (14.9%)	78.7	
**Sex**							1 (7.1%)	32.5	
Female	509 (9.7%)	69.9	1983 (37.7%)	71.6	1915 (36.4%)	76.7			
Male	897 (10.2%)	66.7	3499 (39.8%)	68.8	3136 (35.7%)	73.2	847 (16.1%)	80.4	
**Marital status^b^**									
Never married	227 (12.7%)	60.6	785 (43.8%)	62.5	562 (31.4%)	67.9	217 (12.1%)	71.6	
Married	708 (8.9%)	67.9	2999 (37.7%)	70.1	2937 (36.9%)	74.4	1305 (16.4%)	78	
Widowed/Divorced/Separated	405 (10.8%)	72.1	1476 (39.2%)	73.1	1376 (36.5%)	77.6	508 (13.5%)	81.7	
**Histology of RCC**									
ccRCC	695 (10.1%)	67.4	2801 (40.6%)	69.7	2511 (36.4%)	74.4	900 (13.0%)	78.8	
paRCC	197 (12.3%)	68	712 (44.3%)	68.7	537 (33.4%)	74	160 (10.0%)	73.7	
chRCC	49 (10.4%)	72.9	177 (37.7%)	72.6	174 (37.0%)	77.5	70 (14.9%)	85.3	
Undefined RCC	328 (8.3%)	68.1	1339 (33.8%)	69.9	1477 (37.2%)	74.6	822 (20.8%)	77.4	
Other	137 (12.5%)	67.6	453 (41.3%)	70.6	352 (32.1%)	75	156 (14.2%)	79.4	
**Grade of RCC**									
I+II	758 (9.3%)	67.7	3094 (38.0%)	69.9	3050 (37.5%)	74.6	1234 (15.2%)	77.8	
III+IV	389 (12.3%)	67.5	1463 (46.4%)	69.6	1016 (32.2%)	73.7	287 (9.1%)	77.3	
Unknown	259 (9.4%)	68.9	925 (33.6%)	69.7	985 (35.7%)	75.3	587 (21.3%)	78.6	
**Laterality^b^**									
Left	688 (10.1%)	67.8	2656 (39.0%)	69.8	2423 (35.6%)	74.4	1039 (15.2%)	78.7	
Right	716 (9.9%)	68	2826 (39.1%)	69.8	2626 (36.3%)	74.7	1068 (14.8%)	77.2	
**Surgery**									
Partial nephrectomy	314 (10.0%)	66.5	1273 (40.6%)	68.5	1148 (36.6%)	73.3	400 (12.8%)	77.7	
Radical nephrectomy	1092 (10.0%)	68.3	4209 (38.6%)	70.2	3903 (35.8%)	74.9	1708 (15.6%)	78	
**Tumor size group**									
~ 2 cm	191 (11.0%)	64.1	658 (37.8%)	67	609 (35.0%)	73.2	282 (16.2%)	78.9	
2–3 cm	251 (8.6%)	67.3	1101 (37.7%)	69.8	1094 (37.5%)	75	471 (16.1%)	77.2	
3–4 cm	317 (10.1%)	68.6	1181 (37.5%)	70.1	1163 (36.9%)	75.2	492 (15.6%)	78.4	
4–5 cm	272 (10.1%)	69.2	1057 (39.2%)	70.4	989 (36.7%)	74.8	379 (14.1%)	76.5	
5–7 cm	375 (10.6%)	68.6	1485 (41.9%)	70.3	1196 (33.8%)	73.9	484 (13.7%)	78.3	
**Second primary malignancy onset**									
Yes	215 (5.9%)	68.3	1291 (35.5%)	70.9	1456 (40.0%)	74.4	678 (18.7%)	76.9	
No	1191 (11.4%)	67.8	4191 (40.3%)	69.5	3595 (34.5%)	74.6	1430 (13.7%)	78.5	

ccRCC, clear cell renal cell carcinoma; paRCC, papillary renal cell carcinoma; chRCC, chromophobe renal cell carcinoma.

a, % of total death.; b, not present the information of unknown race (n = 536), unknown the Marital status (n = 3198), and unknown laterality (n = 17).

### Standardized Mortality Ratios

**Figure 3:**
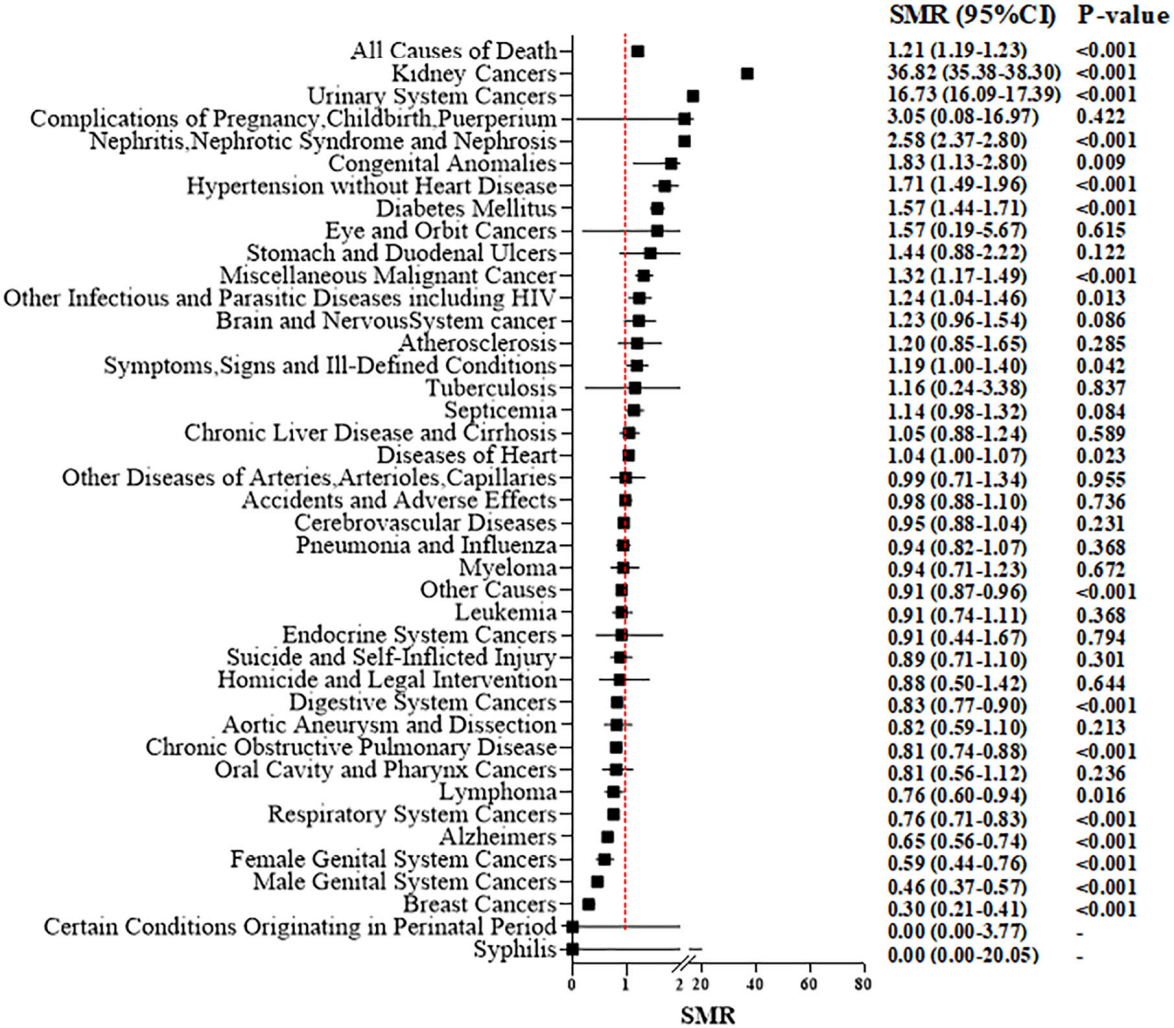
Standardized mortality ratios (SMR) for each causes of death after T1N0M0 renal cell carcinoma cancer diagnosis.

**Figure 4:**
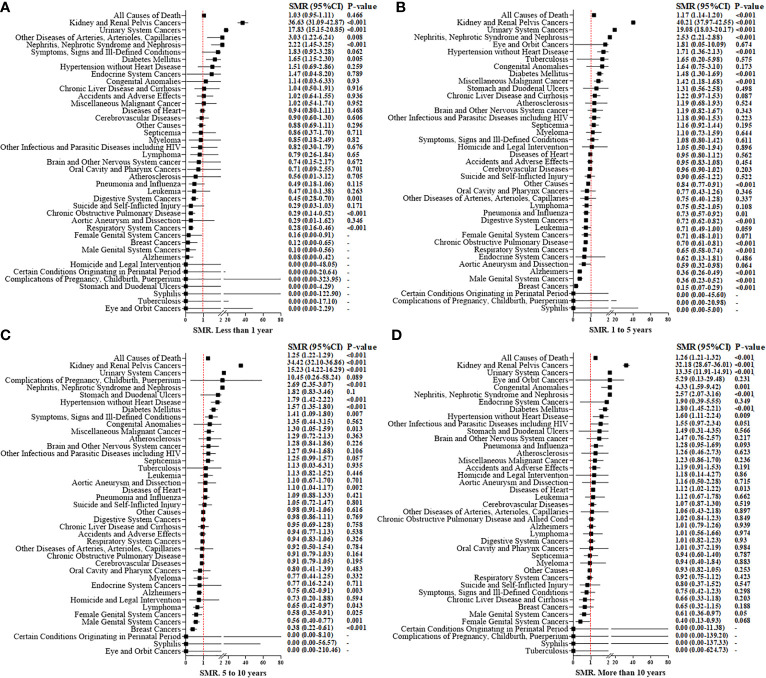
Standardized mortality ratios (SMR) for causes of death in each latency period of < 1 year **(A)**, 1 to 5 years **(B)**, 5 to10 years **(C)**, and more than 10 years **(D)** after T1N0M0 renal cell carcinoma cancer diagnosis.

The overall SMR showed a 21% increased mortality (SMR, 1.21; 95%CI 1.19–1.23; *P* < 0.001, **Figure 3**). With an increase in time intervals, the SMR increased slightly. For people who died <1 year after the RCC diagnosis, the overall SMR showed no significant difference when comparing RCC patients and the general population (SMR, 1.03; 95%CI 0.95–1.11; *P* = 0.466). RCC patients who died within 5 years after treatment did not show a higher risk of mortality due to heart disease compared with that in the general population **Figures 4A, B**). However, for RCC patients who died >5 years after the diagnosis, the SMR for heart disease increased significantly (**Figures 4C, D**).

For patients who underwent PN, the overall SMR showed no significant difference compared with that in the general population (SMR: 1.02; 95%CI 0.98–1.06; *P* = 0.327) (**Supplementary Figure 2A**). However, for RN patients, a higher overall risk of mortality (1.27; 95%CI 1.25–1.30; *P* < 0.001) (**Supplementary Figure 2B**) compared with that in the general population was suggested. For death due to non-cancer-related disease, we discovered that nephritis, nephrotic syndrome, and nephrosis (SMR, 2.58; 95%CI, 2.37–2.80; *P* < 0.001), hypertension (1.71; 1.49–1.96; <0.001), heart diseases (1.04; 1.00–1.07; 0.023) and diabetes mellitus (1.57; 1.4–1.71; <0.001) showed a significantly high SMR (especially for RN patients) compared with that for the general population. But, for patients who underwent PN, there was no significant increase in death due to hypertension or heart diseases.

**Supplementary Figure 3** shows the age-specific SMR and stratification by the type of surgical procedure. Compared with the corresponding general population of identical age group, we discovered a 2.49-fold increase in overall mortality among younger (~49 years) RCC patients (SMR, 2.49; 95%CI, 2.27–2.73; *P* < 0.001), which then decreased steadily with increasing age (SMR = 1.84 for 50–59 years, 1.49 for 60–69 years, and 1.04 for 70+ years; *P* < 0.001 for all). These trends were consistent with the observation in RN-treated patients, with a SMR of 2.88 for those aged ~49 years, 2.06 for people aged 50–59 years, 1.66 for individuals aged 60–69 years, and 1.08 for those aged 70+ years (*P* < 0.001 for all). These trends were also consistent with the observation in PN-treated patients, with a SMR of 1.78 for those aged ~49 years, 1.39 for people aged 50–59 years, 1.14 for individuals aged 60–69 years, and 0.91 for those aged 70+ years (*P* < 0.001 for all). For patients aged >70 years, death due to cerebrovascular causes (SMR, 0.91; *P* < 0.001) and mortality due to heart disease (0.86; <0.001) in the RCC cohort was lower than that in the general population. Irrespective of age and type of surgical procedure, the risk of death from nephritis, nephrotic syndrome, or nephrosis was increased significantly for RCC patients after surgical treatment compared with that in the general population.

### Risks Factors of All-Cause, and Different Causes, of Mortality

**Table 3** presents the risk factors for each cause of mortality. CVD-specific mortality was strongly associated with age (50–59 years *vs.* ~49 years: adjusted HR, 2.24; 60–69 years *vs.* ~49 years: adjusted HR, 4.14; 70+ years *vs.* ~49 years, adjusted HR, 9.28; *P* < 0.001 for all). An increased risk of CVD-related death was observed for black patients compared with that in white patients (adjusted HR, 1.62; 95%CI, 1.48–1.77; *P* < 0.001), but this was not positively associated with RCC-related mortality (adjusted HR, 0.94; 95%CI, 0.82–1.07; *P* = 0.320). Moreover, other races showed a lower risk of mortality due to CVD compared with that in white patients. As well as male patients, patients who were widowed/divorced/separated, patients who underwent PN, and patients without a second primary malignancy carried a higher risk of CVD- or RCC-related mortality. Tumor diameter was significantly associated with RCC-related mortality (adjusted HR = 1.24 for 2–3 cm *vs.* ~2 cm: 1.72 for 3–4 cm *vs.* ~2 cm: 2.64 for 4–5 cm *vs.* ~2 cm: 4.14 for 5–7 cm *vs.* ~2 cm), but not for CVD-related mortality. A tumor grade of III/IV compared with I/II suggested a higher risk of death from RCC (adjusted HR, 2.18; 95%CI, 2.00–2.37, *P* < 0.001) and lower risk of CVD-specific mortality (0.88; 0.81–0.96; 0.003). Chromophobe RCC showed a lower risk of RCC- and CVD-related mortality. RN was significantly associated with each specific mortality compared with PN (HR for other non-cancer-related diseases = 1.63; HR for other cancer types = 1.21; HR for other causes = 1.30; *P* < 0.001 for all). **Supplementary Table 4** suggests that, as the tumor diameter and age of a patient increased, the relative risk of CVD between RN and PN decreased gradually. In RCC tumors of diameter >4 cm, there was no significant difference in risk of death from CVD between PN and RN (adjusted HR, 1.01; 95%CI, 0.79–1.29; *P* = 0.950).

**Table 3:** Hazard ratio and 95% confidence intervals for predicting all mortality and different causes mortality amongT1N0M0 renal cell carcinoma patients.

	All mortality#	Causes of mortality¶				
		Renal cell carcinoma	Cardiovascular disease	Other non-cancer diseases	Other cancers	Non-diseases cause
	Adjusted	Adjusted	Adjusted	Adjusted	Adjusted	Adjusted
	HR (95%CI)	sHR (95%CI)	sHR (95%CI)	sHR (95%CI)	sHR (95%CI)	sHR (95%CI)
**Year group of diagnosis**						
2000-2004	1 reference	1 reference	1 reference	1 reference	1 reference	1 reference
2005-2009	0.94 (0.89-0.98) *	0.78 (0.70-0.88) *	0.67 (0.61-0.74) *	0.72 (0.65-0.80) *	0.78 (0.69-0.88) *	0.82 (0.73-0.93) *
2010-2015	0.89 (0.83-0.96) *	0.33 (0.28-0.39) *	0.26 (0.23-0.30) *	0.25 (0.21-0.30) *	0.38 (0.31-0.46) *	0.30 (0.25-0.36) *
**The age group of diagnosis**						
</=49 years	1 reference	1 reference	1 reference	1 reference	1 reference	1 reference
50-59 years	1.83 (1.71-1.96) *	1.66 (1.44-1.92) *	2.29 (1.97-2.65) *	1.94 (1.66-2.27) *	1.76 (1.46-2.12) *	1.18 (1.00-1.38) *
60-69 years	3.16 (2.96-3.38) *	2.37 (2.06-2.72) *	4.14 (3.60-4.77) *	3.17 (2.73-3.67) *	2.59 (2.16-3.09) *	2.00 (1.73-2.31) *
70+ years	6.78 (6.36-7.23) *	3.04 (2.64-3.49) *	9.28 (8.10-10.63) *	5.82 (5.05-6.71) *	4.15 (3.48-4.96) *	3.97 (3.46-4.56) *
**Race**						
White	1 reference	1 reference	1 reference	1 reference	1 reference	1 reference
Black	1.30 (1.23-1.37) *	0.94 (0.82-1.07)	1.62 (1.48-1.77) *	1.51 (1.36-1.67) *	0.80 (0.70-0.92) *	1.08 (0.95-1.23)
Other	0.94 (0.86-1.01)	1.05 (0.88-1.24)	0.80 (0.68-0.94) *	0.82 (0.68-0.99) *	1.07 (0.89-1.29)	1.07 (0.89-1.29)
**Sex**						
Female	1 reference	1 reference	1 reference	1 reference	1 reference	1 reference
Male	1.35 (1.30-1.40) *	1.28 (1.18-1.39) *	1.44 (1.34-1.54) *	1.17 (1.08-1.27) *	1.11 (1.01-1.21) *	1.35 (1.23-1.48) *
**Marital status**						
Never married	1 reference	1 reference	1 reference	1 reference	1 reference	1 reference
Married	0.71 (0.67-0.75) *	0.98 (0.86-1.11)	0.74 (0.67-0.81) *	0.66 (0.59-0.73) *	0.86 (0.75-0.98) *	0.68 (0.60-0.77) *
Widowed/Divorced/Separated	1.09 (1.03-1.15) *	1.19 (1.03-1.38) *	1.14 (1.03-1.28) *	0.99 (0.88-1.12) *	1.13 (0.97-1.31)	0.91 (0.79-1.05)
**Histology of RCC**						
ccRCC	1 reference	1 reference	1 reference	1 reference	1 reference	1 reference
paRCC	1.03 (0.98-1.09)	0.77 (0.67-0.90) *	1.03 (0.93-1.15)	1.31 (1.16-1.47) *	1.00 (0.87-1.15)	1.09 (0.95-1.26)
chRCC	0.69 (0.63-0.76) *	0.40 (0.30-0.53) *	0.78 (0.67-0.92) *	0.82 (0.67-1.00) *	0.79 (0.62-0.99) *	0.88 (0.71-1.10)
Undefined RCC	1.05 (1.01-1.10) *	1.05 (0.95-1.15)	1.00 (0.92-1.08)	1.10 (1.01-1.21) *	1.12 (1.01-1.24) *	1.18 (1.06-1.30) *
Other	1.27 (1.19-1.35) *	1.39 (1.20-1.60) *	1.07 (0.94-1.21)	1.12 (0.97-1.30)	1.39 (1.19-1.61) *	1.07 (0.90-1.27)
**Grade of RCC**						
I+II	1 reference	1 reference	1 reference	1 reference	1 reference	1 reference
III+IV	1.24 (1.19-1.29) *	2.18 (2.00-2.37) *	0.88 (0.81-0.96) *	1.06 (0.96-1.16)	1.03 (0.92-1.14)	1.00 (0.89-1.11)
Unknown	1.05 (1.00-1.10) *	1.24 (1.11-1.38) *	1.06 (0.98-1.15)	0.91 (0.82-1.00)	1.04 (0.94-1.16)	0.98 (0.88-1.10)
**Laterality**						
Left	1 reference	1 reference	1 reference	1 reference	1 reference	1 reference
Right	1.00 (0.97-1.04)	0.98 (0.90-1.05)	1.05 (0.98-1.11)	1.01 (0.94-1.09)	1.03 (0.95-1.12)	0.91 (0.84-0.99) *
**Surgery**						
Partial nephrectomy	1 reference	1 reference	1 reference	1 reference	1 reference	1 reference
Radical nephrectomy	1.39 (1.33-1.45) *	1.52 (1.35-1.71) *	1.33 (1.22-1.45) *	1.63 (1.47-1.80) *	1.21 (1.09-1.34) *	1.30 (1.17-1.46) *
**Tumor size group**						
~ 2cm	1 reference	1 reference	1 reference	1 reference	1 reference	1 reference
2-3cm	1.00 (0.94-1.06)	1.24 (1.02-1.50) *	0.96 (0.86-1.07)	0.91 (0.80-1.03)	1.12 (0.97-1.29)	0.94 (0.82-1.08)
3-4cm	1.11 (1.05-1.18) *	1.72 (1.43-2.07) *	1.03 (0.92-1.15)	0.94 (0.83-1.07)	1.19 (1.03-1.38) *	0.95 (0.82-1.10)
4-5cm	1.21 (1.14-1.29) *	2.64 (2.20-3.18) *	0.98 (0.87-1.10)	0.92 (0.80-1.05)	1.21 (1.04-1.42) *	1.00 (0.86-1.17)
5-7cm	1.34 (1.26-1.43) *	4.14 (3.46-4.95) *	0.93 (0.83-1.05)	0.79 (0.69-0.91) *	1.26 (1.08-1.46) *	0.94 (0.81-1.09)
**Second primary malignancy onset**						
Yes	1 reference	1 reference	1 reference	1 reference	1 reference	1 reference
No	0.82 (0.79-0.85) *	1.24 (1.12-1.37) *	1.85 (1.69-2.03) *	1.82 (1.63-2.02) *	0.05 (0.05-0.06) *	1.63 (1.45-1.83) *

CI, confidence interval; sHR, Sub-distribution hazard ratio; HR, hazard ratio.

#Cox hazard regression was used to compute the hazard ratio.

¶Fine and Gray model was used for a sub-distribution hazard ratio.

*P value is less than 0.05, which is statistically significant.

## Discussion

With recent progress in the screening, diagnosis, and treatment of many types of cancer, the number of cancer survivors is increasing steadily, and is expected to increase to 20.3 million by 2026 (15). In our study, excellent 10-year RCC-specific survival was observed: 93.5% for patients with T1N0M0 RCC (data not shown). Given the decrease in mortality of T1N0M0 RCC survivors, those patients treated with a curative surgery can expect long-term survival, then the occurrence of competing events such as CVD, other comorbidities, and a second primary malignancy challenges long-term OS and is a concern (16). Our study showed 10-year OS of 72.8% for patients with T1N0M0 RCC (data not shown). Compared with RCC-specific survival of 93.5%, OS decreased substantially due mainly to non-RCC-related deaths, which accounted for ~80% of all-cause mortality in our study. CVD was a significant cause of non-RCC-related death, accounting for 28.3% of all-cause of mortality, and heart disease accounted for 76.2% of all CVD-related death. In fact, CVD has been reported to be the leading cause of death worldwide. An estimated 17.9 million people were reported to have died from CVD in 2016, representing 31% of all global deaths and, of these deaths, 85% were due to heart attack and stroke (17). Cancer survivors might experience an increased risk from CVD due to lifestyle- or cancer treatment-induced damage (18, 19). Taking into account that CVD risk increases with age, the SMR was used to compare cause-specific death with that in the general population of the USA. Overall, we found that, after PN/RN treatment in patients with T1N0M0 RCC, the overall risk of death was increased compared with the corresponding-age general population of the USA. Also, the SMR decreased with age, indicating that age is a crucial prognostic risk factor. We observed that older age and previous RN could significantly increase the risk of all-cause and non-RCC-related mortality. Collectively, non-RCC-related death (especially CVD) was a primary cause of mortality and impacted OS for RCC survivors. Therefore, for the holistic care of RCC survivors, CVD and other non-RCC-specific causes of mortality must be taken into account in the surveillance of survivors post-treatment, especially for older RN patients with comorbidities.

Treatments for local RCC comprise PN, RN, local ablation (e.g., cryosurgery and radiofrequency ablation) and active surveillance (20, 21). PN and RN can achieve curative treatment, and are the main treatment methods in RCC. Given the favorable survival outcomes across PN and RN, preservation of kidney function and minimizing surgery-related complications are often of paramount concern, which is important for clinical decision-making. PN is recommended by international guidelines in preference to RN whenever technically feasible (22). PN not only allows patients to obtain oncology control comparable with that achieved with RN, it also retains kidney function effectively. Compared with RN, PN has been shown to provide improved kidney function (23), a lower risk of CVD, and improved OS (24–27). We demonstrated that, compared with patients who underwent RN, the cumulative mortality incidence by CVD or RCC in patients treated by PN was lower. A randomized trial by the European Organisation for Research and Treatment of Cancer compared the oncologic outcome of elective PN *versus* RN for low-stage RCC. They demonstrated no difference in cancer-specific survival and did not show RN to be associated with an increased risk of death for non-cancer-related causes (and especially cardiovascular events) compared with PN (28). Miller et al. conducted a retrospective registry-based cohort study using SEER–Medicare data on RCC patients who underwent PN/RN. They did not observe an association between treatment and postoperative cardiovascular morbidity (29). Miller and colleagues included only RCC patients aged >66 years, and the study period was between 2000 and 2002; only 7% patients underwent PN (compared with 40.8% cases who had PN in our study), and regional invasion of RCC was included in their cohort.

We found that, compared with PN, RN carried a significantly higher risk of CVD and an increased incidence of CVD, especially for small RCCs (diameter <2 cm). Our results are consistent with data from other studies (24, 27, 30). Nevertheless, with increasing tumor diameter (>4 cm), the surgical approach was not a significant predictor of CVD. This observation suggests that, although PN had a superior benefit in terms of preservation of kidney function compared with that achieved with RN, PN may increase the risk of postoperative complications with increasing tumor diameter and RCC complexity. Moreover, in cases where with PN a significant preservation of normal quality renal parenchyma could not be warranted, any benefit in terms of kidney function and CVD control may be reduced (31, 32).

Due to the complications and competing events induced by surgical interventions and the potential for overdiagnosis and overtreatment of localized small kidney masses, active surveillance has gained acceptance gradually as a management alternative to surgery and focal therapy for localized small RCC (20, 33). Although data from randomized clinical trials are not available, active surveillance is a safe initial management strategy for small local RCC, especially for RCC patients with tumors of very small diameter (<2 cm), older patients (>75 years), and/or those who are most ill (34, 35). McIntosh et al. (33) undertook a study with over 5 years of follow-up. They found that active surveillance with or without delayed intervention was a successful strategy for carefully managed older patients with local RCC. Among patients who require delayed intervention after active surveillance, this usually occurs within the first 2–3 years. The prevalence of 5-year cumulative delayed intervention was 42%, and the probability of delayed intervention decreased with time. A low prevalence of RCC-specific death was observed (1.2% at 5 years), so active surveillance appeared safe in the medium-to-long-term. However, Sun and colleagues indicated that patients who underwent RN/PN had a significantly lower risk of death due to RCC compared with that from non-surgical treatment. Also, for patients older than 75 years, there was no significant difference between PN/RN and non-surgical treatment for RCC-specific mortality (35). However, they also found that a group of patients who did not undergo surgical treatment carried a higher risk of dying from other causes than those who underwent surgery. Possible reasons are that patients who were not recommended for surgery and who chose to wait may have had a poor physical performance and could not tolerate surgery. Thus, even without surgery, these patients were not able to avoid death due to other competing health risks from coexisting diseases. Those data suggest that, for older individuals with localized RCC, surgical intervention remains first-line treatment. However, this strategy may not benefit patients with limited life expectancy (especially those with fragile health who cannot tolerate surgery) because the risk of other methods of mortality outweighs the benefits of the RCC-specific survival benefit from surgery (35).

Sun et al. (26) found that increasing age, a higher Charlson Comorbidity Index, being female, hypercalcemia at baseline, hyperlipidemia at baseline, and year of surgery were independent predictors of other causes of mortality. They also reported that, compared with PN-treated patients, RN-treated patients were more likely to die of other causes after surgery, findings that are inconsistent with our results. The importance of comorbidities was underlined by Larcher and colleagues (36), who found that sicker patients with relevant comorbidities benefited most from PN in terms of other-cause mortality. RN increased the risk of other-cause mortality according to an increasing Charlson Comorbidity Index at baseline. Chang and colleagues (37) reported that older age, higher American Society of Anesthesiologists score, and lower body mass index were independent predictors of death from other causes in patients with localized RCC. In their study, of 1,004 patients with T1/2N0M0 RCC, 91.2% were alive, whereas 4.5% and 4.3% had died from RCC and other causes, respectively. CVD, cerebrovascular disease, pulmonary disease, and other malignancies were the most common causes of mortality, and the cumulative incidence of death from other causes increased steadily over time. Kutikov and coworkers (11) described a competing-risks nomogram with the covariables of age, race, sex, and tumor diameter for predicting RCC-specific, non-cancer-related, and other cancer-related death in patients with localized RCC who underwent RCC-directed surgery. Further external validation is needed, as well as an assessment of calibration and discriminatory abilities if the predictions of a model are considered for use in clinical practice.

Full understanding of the causes of death due to local RCC is a prerequisite for follow-up and prognostic evaluation of patients, as well as patient consultation regarding future health risks. In the present study, CVD was the primary competing risk factor for death, and showed an increasing trend with increasing age. Furthermore, the greater the tumor diameter, the higher was the prevalence of RCC-specific mortality. For example, for a tumor diameter >4 cm combined with age <70 years, RCC-related death was the main cause of mortality, exceeding that elicited by CVD. Furthermore, multivariate regression analysis suggested that age and tumor diameter were independent predictors for OS; however, tumor diameter alone was an independent risk factor for RCC-specific death but not CVD. Although tumor diameter cannot predict CVD alone, implementation of RN in small-volume RCC can increase the incidence of CVD. This observation may be because the smaller the tumor volume, the larger the volume of the normal renal parenchyma. If RN is undertaken in RCC of small diameter, it is equivalent to removal of an entire normal kidney, which causes difficulties in compensation of kidney function, and acts as a further impediment to patients who already have renal insufficiency.

Our study had eight main limitations. First, this was a cohort study using 18 registers. It had the characteristics of a retrospective study with the limitations of data heterogeneity and missing data (e.g., basal kidney function, comorbidities). Second, mortality by CVD may have been miscoded. On a death certificate, coronary heart disease may be documented as the cause of death. National mortality statistics based on death-certificate data can overestimate the frequency of coronary heart disease by 7.9%–24.3%. This is especially more common in older people, where it leads to a bias in the analysis (38). Furthermore, even subgroup analysis has been performed in our study, being the US general population median age much younger, it’s likely that the general population age subgroup 50–59 is closer to 50 years old while on the contrary the RCC subgroup closer to 59 years old. This possible bias might significantly influence data and should be concerned. Third, there is an important selection bias between patients who undergo PN and those who undergo RN. For example, it is likely that if a comparison between the tumor diameter after PN *vs.* after RN was available, there would be a significant difference. Also, we did not use statistical approaches to reduce biases in selection and unmeasured variables. Therefore, the better oncological results obtained with PN must be interpreted with caution. Fourth, inclusion of patients over a long time in the SEER database could have led to an apparently higher risk of death. Simultaneously, over such a long-time span, many changes may have taken place in terms of cancer treatment and the clinical characteristics of tumors. Patients diagnosed in recent years have a short duration of follow-up, so the chance of dying from any cause is low. We tried to correct these limitations by studying the more modern treatment schedule of 2000–2015. Fifth, considering that use of kidney cancer-targeted drugs can increase CVD risk (19), and to avoid the impact of other tumor radiotherapies and chemotherapies, we selected people with a first diagnosis of local disease with no involvement of lymph nodes or distant metastasis and primary RCC. However, we did not take into account treatment of patients with second primary cancers, which may have impacted our results. Nonetheless, our data showed that the onset of a second primary cancer decreased the CVD risk, which suggests that the two events exist as competing relationships. Sixth, it has been acknowledged that the SEER database does not provide information concerning the comorbidities and health status of patients, which increased the risk of non-RCC-related mortality. We did not use the registry linked to Medicare claims, which provides information about comorbidities. Although we used the SMR and compared it with that in the general population, most patients with RCC might have significantly more comorbidities compared with those in the general population. Therefore, a study is needed to compare mortality prevalence with those who had surveillance or observation of their kidney masses. Seventh, there was no adjustment of the SMR for other confounding factors (as in a multivariate regression analysis), nor did we consider the time-to-event risk (as in Cox’s proportional hazard regression). Therefore, other risk factors besides age may have affected our results, and multivariate analysis of RCC patients and non-RCC cohorts will be required in the future. Eighth, overall, the socioeconomic status of the population covered by the SEER database is low, and the diversity of ethnic minorities is high. In addition, the SEER database identified just 28% of all cancer cases, which may invoke a major bias in data submission, and means that our results may not represent the entire USA population or other populations.

## Conclusions

We described the incidence and prognostic factors of mortality due to RCC and other non-RCC-related causes based on a large, population-based cohort. Patients with local RCC had excellent OS, but those with high-risk factors had the worst prognosis for non-RCC-related mortality. Our results may help clinicians identify individuals at higher risk of RCC-, and other non-RCC-specific mortality, and provide more “individualized” treatment and holistic management protocols.

## Data Availability Statement

Publicly available datasets were analyzed in this study. These data can be found here: https://seer.cancer.gov/.

## Author Contributions

ZW, JW, and XZ conceived the research and wrote the manuscript. ZW was in charge of the registry of the the data. ZW, JW, and YZ analyzed the data and prepared the figures and tables. All authors contributed to the article and approved the submitted version.

## Funding

Supported by the National Natural Science Foundation of China (grant 31570988).

## Conflict of Interest

The authors declare that the research was conducted in the absence of any commercial or financial relationships that could be construed as a potential conflict of interest.
