# Supplementary material for: Cause-Specific Mortality Among Survivors From T1N0M0 Renal Cell Carcinoma: A Registry-Based Cohort Study
Source: Front Oncol. 2021 Mar 10;11:604724. doi: 10.3389/fonc.2021.604724 (PMC7988093; doi:10.3389/fonc.2021.604724)
Supplement: Supplementary file 1 [file DataSheet_1.docx]

| Supplementary Table 1. Definition of each cause of death and corresponding codes in the ICD-10 of diseases | | |
| --- | --- | --- |
|  | **ICD-10** | **Cancer Causes of Death** |
| **Cancer Causes of Death** |  |  |
| All Malignant Cancers | C00-C97 |  |
| Oral Cavity and Pharynx |  |  |
|  | C00 | Lip |
|  | C01-C02 | Tongue |
|  | C07-C08 | Salivary Gland |
|  | C04 | Floor of Mouth |
|  | C03, C05-C06 | Gum and Other Mouth |
|  | C11 | Nasopharynx |
|  | C09 | Tonsil |
|  | C10 | Oropharynx |
|  | C12-C13 | Hypopharynx |
|  | C14 | Other Oral Cavity and Pharynx |
| Digestive System |  |  |
|  | C15 | Esophagus |
|  | C16 | Stomach |
|  | C17 | Small Intestine |
| Colon and Rectum |  |  |
|  | C18, C26.0 | Colon excluding Rectum |
|  | C19-C20 | Rectum and Rectosigmoid Junction |
|  | C21 | Anus, Anal Canal, and Anorectum |
| Liver and Intrahepatic Bile Duct |  |  |
|  | C22.0, C22.2-C22.4, C22.7, C22.9 | Liver |
|  | C22.1 | Intrahepatic Bile Duct |
|  | C23 | Gallbladder |
|  | C24 | Other Biliary |
|  | C25 | Pancreas |
|  | C48.0 | Retroperitoneum |
|  | C45.1, C48.1-C48.2 | Peritoneum, Omentum, and Mesentery |
|  | C26.8-C26.9, C48.8 | Other Digestive Organs |
| Respiratory System |  |  |
|  | C30-C31 | Nose, Nasal Cavity and Middle Ear |
|  | C32 | Larynx |
|  | C34 | Lung and Bronchus |
|  | C38.4, C45.0 | Pleura |
|  | C33, C38.1-C38.3, C38.8, C39 | Trachea, Mediastinum and Other Respiratory Organs |
|  | C40-C41 | Bones and Joints |
|  | C47, C49, C38.0, C45.2 | Soft Tissue including Heart |
| Skin & |  |  |
|  | C43 | Melanoma of the Skin |
|  | C44, C46 | Non-Melanoma Skin & |
|  | C50 | Breast |
| Female Genital System |  |  |
|  | C53 | Cervix Uteri |
| Corpus and Uterus, NOS |  |  |
|  | C54 | Corpus Uteri |
|  | C55 | Uterus, NOS |
|  | C56 | Ovary |
|  | C52 | Vagina |
|  | C51 | Vulva |
|  | C57-C58 | Other Female Genital Organs |
| Male Genital System |  |  |
|  | C61 | Prostate |
|  | C62 | Testis |
|  | C60 | Penis |
|  | C63 | Other Male Genital Organs |
| Urinary System |  |  |
|  | C67 | Urinary Bladder |
|  | C64-C65 | Kidney and Renal Pelvis |
|  | C66 | Ureter |
|  | C68 | Other Urinary Organs |
|  | C69 | Eye and Orbit |
|  | C70, C71, C72 | Brain and Other Nervous System |
| Endocrine System |  |  |
|  | C73 | Thyroid |
|  | C37, C74-C75 | Other Endocrine including Thymus $ |
| Lymphoma |  |  |
|  | C81 | Hodgkin Lymphoma |
|  | C82–86, C96.3 | Non-Hodgkin Lymphoma |
|  | C90.0, C90.2, C90.3 | Myeloma |
| Leukemia |  |  |
| Lymphocytic Leukemia |  |  |
|  | C91.0 | Acute Lymphocytic Leukemia |
|  | C91.1 | Chronic Lymphocytic Leukemia |
|  | C91.2-C91.4, C91.6-C91.9 | Other Lymphocytic Leukemia |
| Myeloid and Monocytic Leukemia |  |  |
|  | C92.0, C92.4-C92.6, C92.8, C94.0, C94.2 | Acute myeloid |
|  | C93.0 | Acute Monocytic Leukemia |
|  | C92.1 | Chronic Myeloid Leukemia |
|  | C92.2-C92.3, C92.7, C92.9, C93.1-C93.3, C93.7, C93.9 | Other Myeloid/Monocytic Leukemia |
| Other Leukemia |  |  |
|  | C94.4, C94.5, C95.0 | Other Acute Leukemia |
|  | C90.1, C91.5, C94.1, C94.3, C94.7, C95.1, C95.2, C95.7, C95.9 | Aleukemic, subleukemic and NOS |
|  | C45 | Mesothelioma (ICD-10 only) + |
|  | C46 | Kaposi Sarcoma (ICD-10 only) + |
|  | C26.1, C45.7, C45.9, C76-C80, C88, C94.6, C96.0-C96.2, C96.4-C96.9, C97 | Miscellaneous Malignant Cancer |
| **Non-Cancer Causes of Death** |  |  |
| In situ, benign or unknown behavior neoplasms | D00-D09 | In situ neoplasms |
|  | D10-D36 | Benign neoplasms |
|  | D37-D48 | Neoplasms of uncertain or unknown behavior |
| Septicemia | A40-A41 | Sepsis |
| Other Infectious Diseases |  |  |
| Diabetes Mellitus | E10-E14 | Diabetes mellitus |
| Alzheimer’s | G30 | Alzheimer disease |
| Diseases of Heart | I00-I02 | Acute rheumatic fever |
|  | I05-I09 | Chronic rheumatic heart diseases |
|  | I11 | Hypertensive heart disease |
|  | I13 | Hypertensive heart and renal disease |
|  | I20-I25 | Ischemic heart diseases |
|  | I26-I28 | Pulmonary heart disease and diseases of pulmonary circulation |
|  | I30-I32 | Diseases of pericardium |
|  | I33 | Acute and subacute endocarditis |
|  | I34-I39 | Nonrheumatic valve disorders |
|  | I40-I41 | Myocarditis |
|  | I42-I43 | Cardiomyopathy |
|  | I44-I45 | Conduction disorders |
|  | I46 | Cardiac arrest |
|  | I47-I49 | Arrythmias |
|  | I50 | Heart failure |
|  | I51 | Complications and ill-defined descriptions of heart disease |
| Hypertension without Heart Disease | I10 | Essential (primary) hypertension |
|  | I12 | Hypertensive renal disease |
| Cerebrovascular Diseases | I60-I62 | Nontraumatic intracranial hemorrhage |
|  | I63 | Cerebral infarction |
|  | I64 | Stroke, not specified as hemorrhage or infarction |
|  | I65-I66 | Occlusion and stenosis of precerebral/cerebral arteries, not resulting in cerebral infarction |
|  | I67-I69 | Other cerebrovascular diseases or Sequelae of cerebrovascular disease |
| Atherosclerosis | I70 | Atherosclerosis |
| Aortic Aneurysm and Dissection | I71 | Aortic Aneurysm and Dissection |
| Other Diseases of Arteries, Arterioles, Capillaries | I72-I73 | Other aneurysm and dissection or other peripheral vascular diseases |
|  | I74 | Arterial embolism and thrombosis |
|  | I77 | Other disorders of arteries and arterioles |
|  | I78 | Diseases of capillaries |
| Pneumonia and Influenza | J09-J18 | Influenza and pneumonia |
| Chronic Obstructive Pulmonary Disease | J40-J42 | Bronchitis |
|  | J43 | Emphysema |
|  | J44 | Other chronic obstructive pulmonary disease |
|  | J45-J46 | Asthma or Status asthmaticus |
|  | J47 | Bronchiectasis |
| Chronic Liver Disease and Cirrhosis | K70 | Alcoholic liver disease |
|  | K73 | Chronic hepatitis |
|  | K74 | Fibrosis and cirrhosis of the liver |
| Nephritis, Nephrotic Syndrome and Nephrosis | N00-N07 | Glomerular diseases |
|  | N17-N19 | Renal failure |
|  | N25 | Disorders resulting from the impaired renal tubular function |
|  | N26 | Unspecified contracted kidney |
|  | N27 | The small kidney of unknown cause |
| Symptoms, Signs and Ill-Defined Conditions | R00-R99 | Symptoms, signs, abnormal results of clinical or other investigative procedures, and ill-defined conditions regarding which no diagnosis classifiable elsewhere is recorded. |
| Accidents and Adverse Effects | V01-V99 | Transport accidents |
|  | W00-X59 | Other external causes of accidental injury |
|  | Y85-Y86 | Sequelae of transport accidents or other accidents |
| Suicide and Self-Inflicted Injury | X60-X84 | Intentional self-harm |
|  | Y87 | Sequelae of intentional self-harm, assault, and events of undetermined intent |
| Other Cause of Death |  |  |

| Supplementary Table 2. Cumulative cause-specific mortality by age at diagnosis and treatment. | | | | | |
| --- | --- | --- | --- | --- | --- |
| **Cohort** | **Causes of mortality** | | | | |
|  | **RCC** | **CVD*** | **Other non-cancer diseases** | **Other cancers** | **Other causes/unknown** |
| **PN group** |  |  |  |  |  |
| 5-yr. | 0.9% | 1.7% | 1.0% | 1.0% | 1.0% |
| 10-yr. | 1.4% | 2.9% | 1.9% | 1.8% | 1.8% |
| 15-yr. | 1.5% | 3.4% | 2.1% | 2.1% | 2.0% |
| **RN group** |  |  |  |  |  |
| 5-yr. | 3.1% | 3.6% | 2.5% | 1.9% | 1.9% |
| 10-yr. | 4.8% | 6.3% | 4.6% | 3.5% | 3.5% |
| 15-yr. | 5.4% | 7.4% | 5.5% | 4.2% | 4.1% |
| **~ 49 years old group** |  |  |  |  |  |
| 5-yr. | 0.9% | 0.8% | 0.7% | 0.4% | 1.0% |
| 10-yr. | 1.5% | 1.2% | 1.3% | 0.7% | 1.6% |
| 15-yr. | 1.7% | 1.5% | 1.5% | 0.9% | 1.7% |
| **50-59 years old group** |  |  |  |  |  |
| 5-yr. | 1.7% | 1.7% | 1.4% | 0.9% | 1.1% |
| 10-yr. | 2.7% | 2.9% | 2.3% | 1.8% | 1.7% |
| 15-yr. | 3.1% | 3.3% | 2.8% | 2.2% | 2.0% |
| **60-69 years old group** |  |  |  |  |  |
| 5-yr. | 2.3% | 2.8% | 1.9% | 1.7% | 1.4% |
| 10-yr. | 3.7% | 4.7% | 3.4% | 3.2% | 2.6% |
| 15-yr. | 4.2% | 5.5% | 4.0% | 3.8% | 3.0% |
| **70+ years old group** |  |  |  |  |  |
| 5-yr. | 4.2% | 6.2% | 3.7% | 3.4% | 2.9% |
| 10-yr. | 6.0% | 11.5% | 7.2% | 5.8% | 5.6% |
| 15-yr. | 6.4% | 13.5% | 8.7% | 6.5% | 6.8% |
| RCC, renal cell carcinoma; CVD, Cardiovascular disease; PN, partial nephrectomy; RN, radical nephrectomy. *CVD included heart disease, hypertension, cerebrovascular disease, and atherosclerosis/aortic aneurysm and Dissection. | | | | | |

| Supplementary Tables 3. The causes of mortality for T1N0M0 renal cell carcinoma with the treatment of partial/radical nephrectomy. | | | | | | | | | | | | | | | |
| --- | --- | --- | --- | --- | --- | --- | --- | --- | --- | --- | --- | --- | --- | --- | --- |
|  | **All patients** | | | |  | **Treatment group** | | | | | | | | | |
|  |  |  |  |  |  | **Partial nephrectomy** | | | |  | **Radical nephrectomy** | | | | |
| **Causes of mortality** | No. of patients | % of all follow-up events | % of all death | % of each cause |  | No. of patients | % of all follow-up events | % of all death | % of each cause |  | No. of patients | % of all follow-up events | % of all death | % of each cause |  |
| **1.Renal cell carcinoma** |  |  |  |  |  |  |  |  |  |  |  |  |  |  |  |
| ***Total*** | ***2633*** | ***3.8%*** | ***18.7%*** | ***100.0%*** |  | ***427*** | ***1.5%*** | ***13.6%*** | ***100.0%*** |  | ***2206*** | ***5.4%*** | ***20.2%*** | ***100.0%*** |  |
| **2****.Cardiovascular disease** |  |  |  |  |  |  |  |  |  |  |  |  |  |  |  |
| ***Total*** | **3984** | **5.8%** | **28.3%** | **100.0%** |  | **950** | **3.4%** | **30.3%** | **100.0%** |  | **3034** | **7.4%** | **27.8%** | **100.0%** |  |
| Diseases of Heart | 3037 | 4.4% | 21.6% | 76.2% |  | 738 | 2.6% | 23.5% | 77.7% |  | 2299 | 5.7% | 21.1% | 75.8% |  |
| Cerebrovascular Diseases | 595 | 0.9% | 4.2% | 14.9% |  | 141 | 0.5% | 4.5% | 14.8% |  | 454 | 1.1% | 4.2% | 15.0% |  |
| Hypertension without Heart Disease | 223 | 0.3% | 1.6% | 5.6% |  | 43 | 0.2% | 1.4% | 4.5% |  | 180 | 0.4% | 1.6% | 5.9% |  |
| Atherosclerosis/Aortic Aneurysm and Dissection | 129 | 0.2% | 0.9% | 3.2% |  | 28 | 0.1% | 0.9% | 2.9% |  | 101 | 0.2% | 0.9% | 3.3% |  |
| **3.Other non-cancer diseases** |  |  |  |  |  |  |  |  |  |  |  |  |  |  |  |
| ***Total*** | ***2862*** | ***4.0%*** | ***20.5%*** | ***100.0%*** |  | ***604*** | ***2.3%*** | ***19.3%*** | ***100.0%*** |  | ***2258*** | ***5.7%*** | ***20.9%*** | ***100.0%*** |  |
| Nephritis, Nephrotic Syndrome and Nephrosis | 592 | 0.9% | 4.2% | 20.7% |  | 106 | 0.4% | 3.4% | 17.5% |  | 486 | 1.2% | 4.5% | 21.5% |  |
| Diabetes Mellitus | 573 | 0.8% | 4.1% | 20.0% |  | 134 | 0.5% | 4.3% | 22.2% |  | 439 | 1.1% | 4.0% | 19.4% |  |
| Chronic Obstructive Pulmonary Disease and Allied Cond | 567 | 0.8% | 4.0% | 19.8% |  | 107 | 0.4% | 3.4% | 17.7% |  | 460 | 1.1% | 4.2% | 20.4% |  |
| Pneumonia and Influenza | 236 | 0.3% | 1.7% | 8.2% |  | 53 | 0.2% | 1.7% | 8.8% |  | 183 | 0.5% | 1.7% | 8.1% |  |
| Alzheimer | 227 | 0.3% | 1.6% | 7.9% |  | 43 | 0.2% | 1.4% | 7.1% |  | 184 | 0.5% | 1.7% | 8.1% |  |
| Septicemia | 204 | 0.3% | 1.5% | 7.1% |  | 45 | 0.2% | 1.4% | 7.5% |  | 159 | 0.4% | 1.5% | 7.0% |  |
| Chronic Liver Disease and Cirrhosis | 148 | 0.2% | 1.1% | 5.2% |  | 44 | 0.2% | 1.4% | 7.3% |  | 104 | 0.3% | 1.0% | 4.6% |  |
| Symptoms, Signs and Ill-Defined Conditions | 150 | 0.2% | 1.1% | 5.2% |  | 30 | 0.1% | 1.0% | 5.0% |  | 120 | 0.3% | 1.1% | 5.3% |  |
| Other Infectious and Parasitic Diseases including HIV | 139 | 0.2% | 1.0% | 4.9% |  | 35 | 0.1% | 1.1% | 5.8% |  | 104 | 0.3% | 1.0% | 4.6% |  |
| Others | 26 | 0.0% | 0.2% | 0.9% |  | 7 | 0.0% | 0.2% | 1.2% |  | 19 | 0.0% | 0.2% | 0.8% |  |
| **4.Other non-RCC cancers** |  |  |  |  |  |  |  |  |  |  |  |  |  |  |  |
| ***Total*** | ***2299*** | ***3.2%*** | ***16.3%*** | ***100.0%*** |  | ***590*** | ***2.2%*** | ***18.8%*** | ***100.0%*** |  | ***1709*** | ***4.2%*** | ***15.7%*** | ***100.0%*** |  |
| Respiratory system | 634 | 0.9% | 4.5% | 27.6% |  | 165 | 0.6% | 5.3% | 28.0% |  | 469 | 1.2% | 4.3% | 27.4% |  |
| Digestive system | 622 | 0.9% | 4.4% | 27.1% |  | 157 | 0.6% | 5.0% | 26.6% |  | 465 | 1.1% | 4.3% | 27.2% |  |
| Miscellaneous Malignant Cancer | 280 | 0.4% | 2.0% | 12.2% |  | 56 | 0.2% | 1.8% | 9.5% |  | 224 | 0.6% | 2.1% | 13.1% |  |
| Lymphoma/Myeloma/Leukemia | 237 | 0.3% | 1.7% | 10.3% |  | 79 | 0.3% | 2.5% | 13.4% |  | 158 | 0.4% | 1.4% | 9.2% |  |
| Others | 236 | 0.3% | 1.7% | 10.3% |  | 59 | 0.2% | 1.9% | 10.0% |  | 177 | 0.4% | 1.6% | 10.4% |  |
| Male genital system | 84 | 0.1% | 0.6% | 3.7% |  | 20 | 0.1% | 0.6% | 3.4% |  | 64 | 0.2% | 0.6% | 3.7% |  |
| Brain and Other Nervous System | 77 | 0.1% | 0.5% | 3.3% |  | 26 | 0.1% | 0.8% | 4.4% |  | 51 | 0.1% | 0.5% | 3.0% |  |
| Other urinary system | 73 | 0.1% | 0.5% | 3.2% |  | 18 | 0.1% | 0.6% | 3.1% |  | 55 | 0.1% | 0.5% | 3.2% |  |
| Female genital system | 56 | 0.1% | 0.4% | 2.4% |  | 10 | 0.0% | 0.3% | 1.7% |  | 46 | 0.1% | 0.4% | 2.7% |  |
| **5. Non-diseases causes** |  |  |  |  |  |  |  |  |  |  |  |  |  |  |  |
| ***Total*** | ***2269*** | ***3.3%*** | ***16.1%*** | ***100.0%*** |  | ***564*** | ***2.0%*** | ***18.0%*** | ***100.0%*** |  | ***1705*** | ***4.1%*** | ***15.6%*** | ***100.0%*** |  |
| Unknown | 1552 | 2.3% | 11.0% | 68.4% |  | 360 | 1.3% | 11.5% | 63.8% |  | 1192 | 2.9% | 10.9% | 69.9% |  |
| Accidents and Adverse Effects | 358 | 0.5% | 2.5% | 15.8% |  | 100 | 0.4% | 3.2% | 17.7% |  | 258 | 0.6% | 2.4% | 15.1% |  |
| Unknown | 251 | 0.4% | 1.8% | 11.1% |  | 65 | 0.2% | 2.1% | 11.5% |  | 186 | 0.5% | 1.7% | 10.9% |  |
| Suicide and Self-Inflicted Injury | 92 | 0.1% | 0.7% | 4.1% |  | 35 | 0.1% | 1.1% | 6.2% |  | 57 | 0.1% | 0.5% | 3.3% |  |
| Homicide and Legal Intervention | 16 | 0.0% | 0.1% | 0.7% |  | 4 | 0.0% | 0.1% | 0.7% |  | 12 | 0.0% | 0.1% | 0.7% |  |
|  | | | | | | | | | | | | | | | |

| Supplementary Table 4. Subgroup analysis for the impact of different treatments of partial nephrectomy (as a reference) vs. radical nephrectomy in subgroup population on cardiovascular disease mortality CVD of patients with T1N0M0 renal cell carcinoma ǂ | | | | |
| --- | --- | --- | --- | --- |
| **Subgroups** |  | **Adjusted  sHR*/HR# (95%CI)** | **P-value** |  |
| **Model1*** |  |  |  |  |
| **Tumor size group** |  |  |  |  |
| ~ 2cm |  | 1.87 (1.55-2.25) | <0.001 |  |
| 2-3cm |  | 1.26 (1.09-1.47) | 0.002 |  |
| 3-4cm |  | 1.28 (1.08-1.51) | 0.003 |  |
| 4-5cm |  | 1.12 (0.90-1.38) | 0.310 |  |
| 5-7cm |  | 1.01 (0.79-1.29) | 0.950 |  |
| **The age group of diagnosis** |  |  |  |  |
| </=49 years |  | 1.86 (1.32-2.62) | <0.001 |  |
| 50-59 years |  | 1.47 (1.19-1.81) | <0.001 |  |
| 60-69 years |  | 1.33 (1.14-1.56) | <0.001 |  |
| 70+ years |  | 1.21 (1.07-1.36) | 0.002 |  |
| **Model 2#** |  |  |  |  |
| **Tumor size group** |  |  |  |  |
| ~ 2cm |  | 1.95 (1.63-2.32) | <0.001 |  |
| 2-3cm |  | 1.29 (1.12-1.49) | <0.001 |  |
| 3-4cm |  | 1.26 (1.08-1.49) | 0.004 |  |
| 4-5cm |  | 1.11 (0.90-1.37) | 0.339 |  |
| 5-7cm |  | 0.98 (0.77-1.24) | 0.841 |  |
| **The age group of diagnosis** |  |  |  |  |
| </=49 years |  | 1.84 (1.34-2.54) | <0.001 |  |
| 50-59 years |  | 1.46 (1.20-1.77) | <0.001 |  |
| 60-69 years |  | 1.34 (1.16-1.56) | <0.001 |  |
| 70+ years |  | 1.23 (1.10-1.39) | <0.001 |  |
| CI, confidence interval; sHR, Sub-distribution hazard ratio; HR, hazard ratio. *Fine and Gray model was used for the sub-distribution hazard ratio ＃ Cause-specific hazard regression was used to compute the hazard ratios ǂ All other covariables were adjusted in the multivariate regression analysis. | | | | |
