# Supplementary material for: Cause-Specific Mortality Among Survivors From T1N0M0 Renal Cell Carcinoma: A Registry-Based Cohort Study
Source: Front Oncol. 2021 Mar 10;11:604724. doi: 10.3389/fonc.2021.604724 (PMC7988093; doi:10.3389/fonc.2021.604724)
Supplement: Supplementary file 2 [file DataSheet_2.docx]

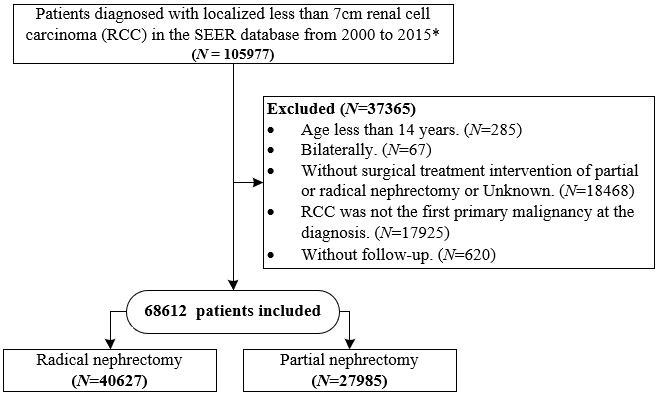


**Supplementary Figure 1.** Flow chart for the data screening.

* There was no information about the tumor-lymphnode-metastasis (TNM) stage before 2004 for renal cell carcinoma in the SEER dataset, and we just included localized and tumor size was less than 7cm, which is equal to T1N0M0 in the current TNM staging system.


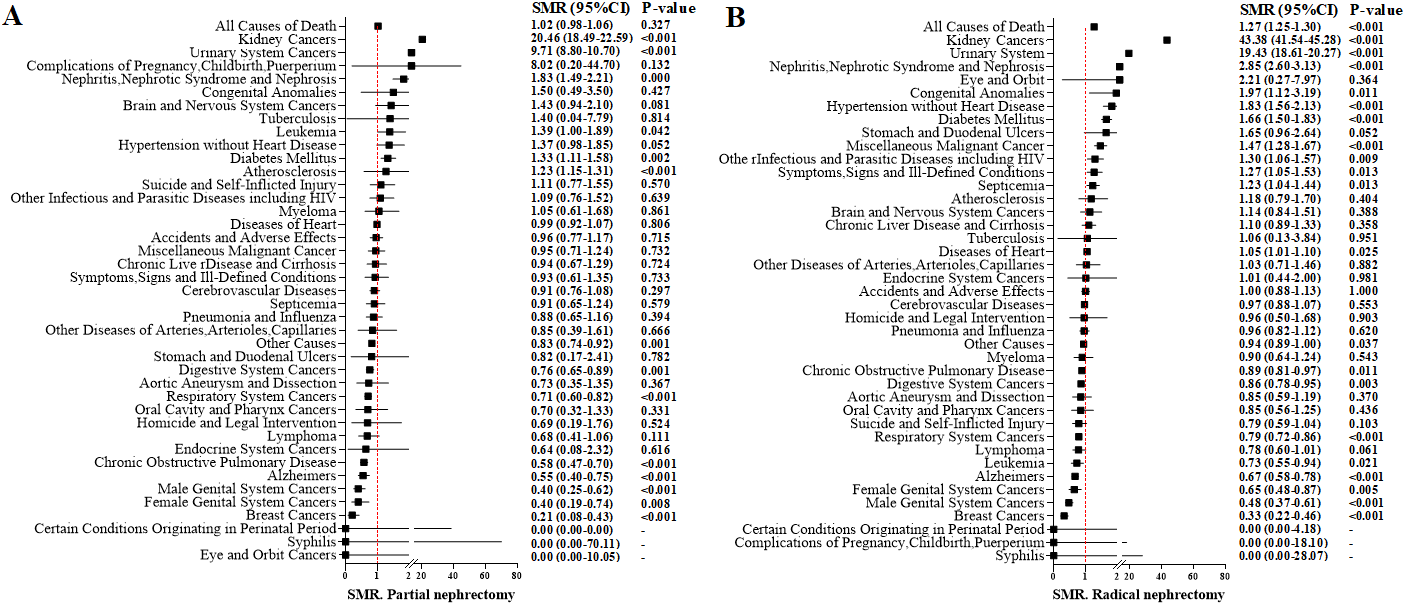


**Supplementary** **Figure 2.** Standardized mortality ratios (SMR) for causes of death based on the treatment after renal cell carcinoma cancer diagnosis


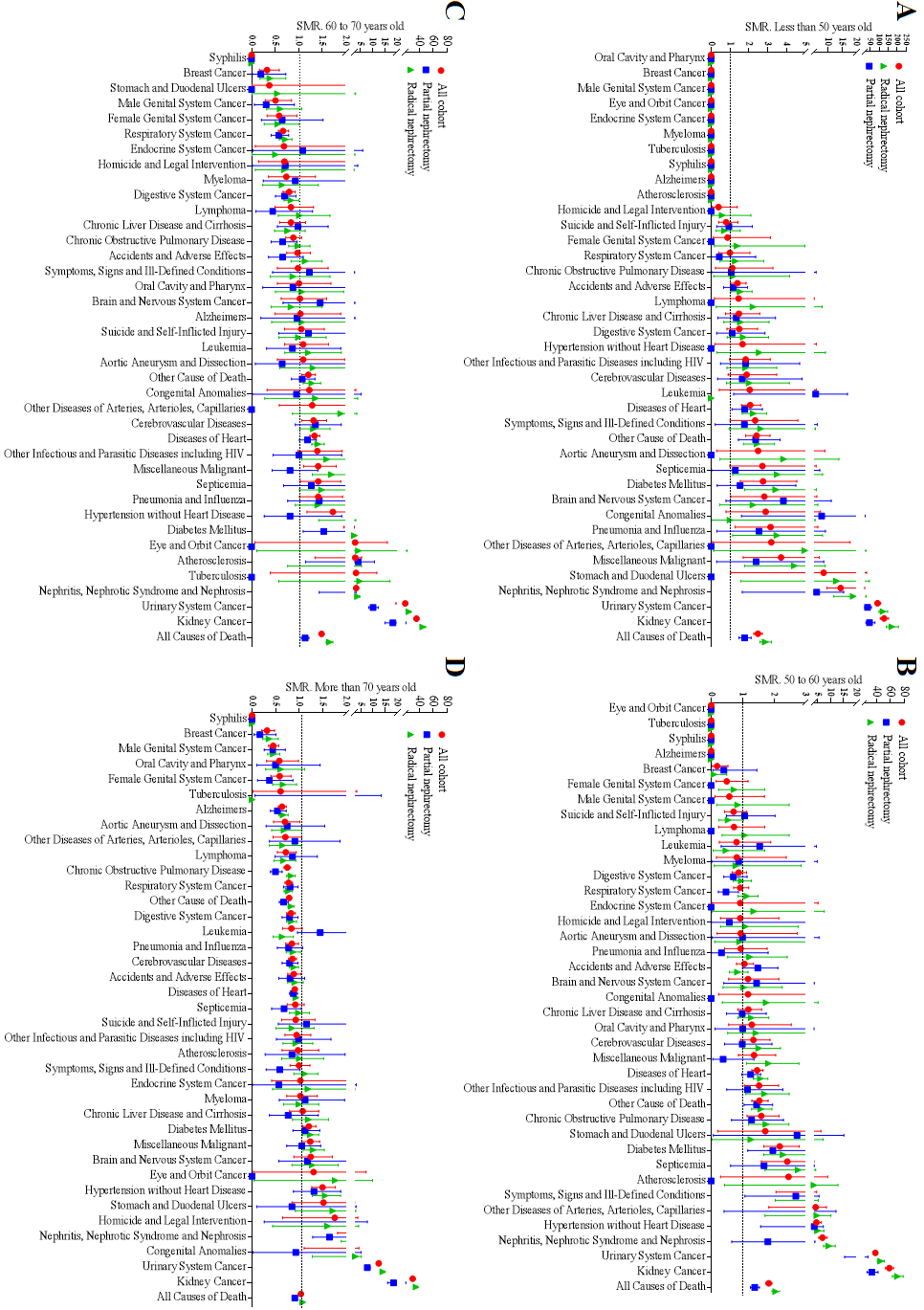


**Supplementary Figure 3.** Age specific standardized mortality ratios (SMR) for each cause of death after renal cell carcinoma cancer diagnosis
